# Hedgehog Interacting Protein (Hhip) Regulates Insulin Secretion in Mice Fed High Fat Diets

**DOI:** 10.1038/s41598-019-47633-3

**Published:** 2019-08-01

**Authors:** Henry Nchienzia, Min-Chun Liao, Xin-Ping Zhao, Shiao-Ying Chang, Chao-Sheng Lo, Isabelle Chenier, Julie R. Ingelfinger, John S. D. Chan, Shao-Ling Zhang

**Affiliations:** 1 Université de Montréal, Centre de recherche du Centre hospitalier de l’Université de Montréal (CRCHUM), Tour Viger, 900 rue Saint-Denis, Montréal, QC H2X 0A9 Canada; 2 Harvard Medical School, Pediatric Nephrology Unit, Massachusetts General Hospital, 55 Fruit Street, Boston, MA 02114-3117 USA

**Keywords:** Cell biology, Metabolism

## Abstract

Hedgehog interacting protein (Hhip) is essential for islet formation and beta-cell proliferation during pancreatic development; abnormally elevated Hhip expression has been linked to human pancreatitis. Here, we investigate the role of Hhip in modulating insulin secretion in adult Hhip mice (Hhip +/− vs. Hhip+/+) fed high fat diets (HFD). Both sexes of HFD-Hhip +/+ mice developed impaired glucose intolerance, that was only ameliorated in male HFD-Hhip +/− mice that had high levels of circulating plasma insulin, but not in female HFD-Hhip +/− mice. HFD stimulated Hhip gene expression, mainly in beta cells. Male HFD-Hhip +/+ mice had more large islets in which insulin content was reduced; islet architecture was disordered; and markers of oxidative stress (8-OHdG and Nox 2) were increased. In contrast, male HFD-Hhip +/− mice had more small islets with increased beta cell proliferation, enhanced GSIS, less oxidative stress and preserved islet integrity. *In vitro*, recombinant Hhip increased Nox2 and NADPH activity and decreased insulin-positive beta cells. siRNA-Hhip increased GSIS and abolished the stimulation of sodium palmitate (PA)-BSA on Nox2 gene expression. We conclude that pancreatic Hhip gene inhibits insulin secretion by altering islet integrity and promoting Nox2 gene expression in beta cells in response to HDF-mediated beta cell dysfunction, a novel finding.

## Introduction

The incidence of type 2 diabetes (T2D) will account for approximately 85–90% of all diabetes cases by 2040 and constitutes a major health burden in developed countries^1^. T2D is a metabolic disorder characterized by hyperglycemia associated with obesity-induced peripheral insulin resistance and intrinsic pancreatic islet beta cell dysfunction^2–4^. Long-term complications of T2D may lead to multiple organ dysfunction affecting the heart, kidney, nervous system, eye, artery and blood vessels.

Pancreatic beta cells are primarily responsible for the production and secretion of insulin for the maintenance of metabolic homeostasis^2–4^. In T2D, chronic elevation of free fatty acids causes beta cell dysfunction, reflected by impaired insulin secretory responses to increased glucose levels. Consequently, accumulating free fatty acids and glucose levels in beta cells synergistically up-regulate endogenous triglyceride levels, leading to progressive cell toxicity and increased apoptosis, a metabolic phenomenon termed “gluco-lipotoxicity”^2,3^. A major challenge, given the epidemic of T2D, is to develop more effective preventive and therapeutic strategies based on better understanding of the underlying pathophysiology^1,4,5^.

Hedgehog (Hh) signaling is essential for the development and function of the endocrine and exocrine pancreas^6–9^. 3 secreted Hh ligands, i.e., Sonic (Shh), Indian (Ihh), and Desert (Dhh) bind to the Patched1 membrane receptor, releasing the tonic inhibition of Smoothened, which is responsible for the activation of transcriptional factors of cubitus interruptus homologs (Glis) – e.g., Gli1 and Gli2 (activators) and Gli3 (repressor) – resulting in the transcription of an array of target genes^10–15^. Pancreatic tissue responds to Hh signaling in a dose-dependent manner during pancreatic development^7,9^, and Hh signaling is also required for maintaining adult beta cell function^7^. An increase of Hh signaling in adult beta cells *in vivo* leads to either a loss of beta cell function resulting in decreased insulin production and impaired insulin response to a glucose challenge or even change differentiated beta cells into beta cell-derived undifferentiated tumor cells^7^.

Hedgehog interacting protein (Hhip) originally discovered as a putative antagonist of Hh ligands regulates cell function via either canonical- or non-canonical Hh pathways^10–17^. Hhip encodes a protein of 700 amino acids and is abundantly expressed in vascular endothelial cells-rich tissues, including the pancreas^18,19^. Hhip null mice (Hhip^**−/**−^) display markedly impaired pancreatic islet formation (45% reduction of islet mass with a decrease of beta cell proliferation by 40%)^9^, underscoring the importance of Hhip in normal pancreatic development, though Hhip^−/−^ mice die shortly after birth mainly due to lung defects^9,12^. In addition, pancreatic islets are highly vascularized and contain a structurally and functionally unique cell composition (alpha-, beta-, and delta-cell)^20^. An earlier study showed that low-level Hhip expression could be detected in normal mature pancreas (i.e., in the cytoplasm of islet cells and in blood vessels), but abnormal elevated Hhip gene expression has been linked to human pancreatitis^18^, suggesting that tight regulation of pancreatic Hhip gene expression might be essential for maintaining normal pancreatic function. Moreover, a genome-wide diabetes profiling database (http://diabetes.wisc.edu) revealed that compared to lean animals, *Hhip* mRNA was markedly elevated in the islets of diabetic *ob/ob* mice (at the age of 4 and 10 weeks with both C57BL/6 and BTBR backgrounds), but not in other tissues such as liver, gastrocnemius and soleus muscles, adipose tissue and hypothalamus, underscoring the specificity of *Hhip* expression in murine T2D islets.

Less is known about Hhip expression pattern in mature islet cells and its function under normal and/or stressed conditions. In the present study, we systematically studied the role of pancreatic Hhip expression in response to high fat diet (HFD)-mediated beta cell dysfunction *in vivo*, *ex vivo* and *in vitro*. We showed that HFD-induced pancreatic Hhip gene expression targets beta cells and then inhibits glucose stimulated insulin secretion (GSIS). Mechanistically, Hhip activates oxidative stress-related NADPH oxidase 2 (Nox 2), one of the key factors implicated in beta cell dysfunction^21,22^, impairing insulin secretion and/or action.

## Results

### Metabolic characterization *in vivo*

**Figure 1:**
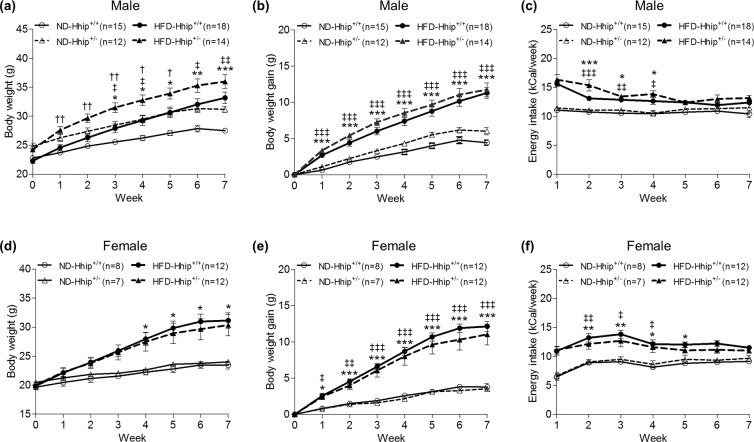
Physiological parameters in both male (a-c, ND-Hhip^+/+^, n = 15; ND-Hhip^+/−^, n = 12; HFD-Hhip^+/+^, n = 18; and HFD-Hhip^+/−^, n = 14) and female (d-f, ND-Hhip^+/+^, n = 8; ND-Hhip^+/−^, n = 7; HFD-Hhip^+/+^, n = 12; and HFD-Hhip^+/−^, n = 12) mice from the age of 6 to 14 weeks. (**a,d**) body weight (BW, **g**); (**b,e**) body weight gain; (**g,c,f**) energy intake calculated based on food consumption per week (kCal/week). Data shown as mean ± SEM; 1 way-ANOVA followed by Bonferroni’s post hoc test. *p ≤ 0.05; **p ≤ 0.01; ***p ≤ 0.001 vs. ND-Hhip^+/+^; ^ǂ^*p* ≤ 0.05; ^ǂǂ^*p* ≤ 0.01; ^ǂǂǂ^*p* ≤ 0.001 vs. ND-Hhip^+/−^; ^†^*p* ≤ 0.05; ^††^*p* ≤ 0.01, HFD-Hhip^+/+^ vs. HFD-Hhip^+/−^.

Heterozygous Hhip (Hhip^+/−^) mice and control littermates (Hhip^+/+^) (Jackson Laboratories) were used [N.B., Adult Hhip^+/–^ mice are phenotypically indistinguishable from control littermates (Hhip^+/+^), whereas Hhip^−/−^ die after birth due to lung defects; thus, Hhip^+/−^ mice were used in the current study^9,12^]. We compared the growth pattern and energy intake of both male and female animals fed either normal diet (ND) or HFD from the age of 6 until 14 weeks (Fig. 1a–c, male; Fig. 1d–f, female). As expected, HFD progressively increased body weight (BW) in both sexes of Hhip^+/+^ vs. Hhip^+/−^ animals (Fig. 1a, male; Fig. 1d, female) with similar BW gain patterns (Fig. 1b, male; Fig. 1e, female). Notably, in both ND and HFD conditions, male Hhip^+/−^ mice were heavier than male Hhip^+/+^ mice, while female animals (Hhip^+/+^ and Hhip^+/−^) had similar BW.

**Figure 2:**
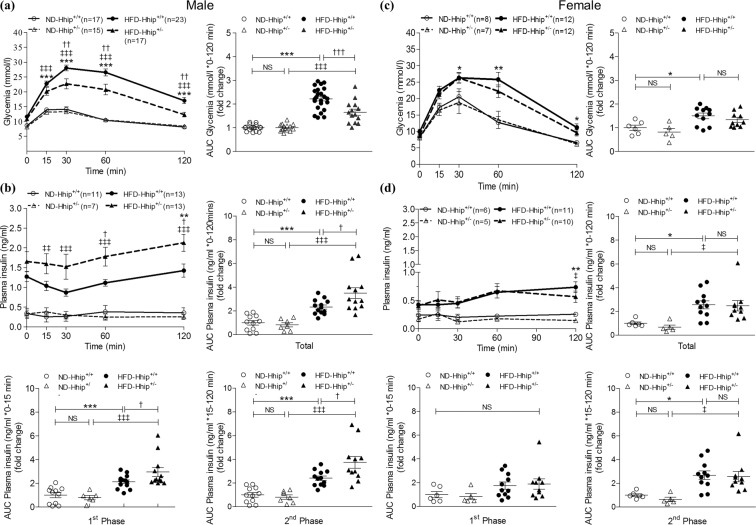
Metabolic parameters in male (**a,b**) and female (**c,d**) mice at the age of 14 weeks. (**a,c**) ipGTT and ipGTT-area under the curve (AUC) quantification (0–120 mins). (**a**: ND-Hhip^+/+^, n = 17; ND-Hhip^+/−^, n = 15; HFD-Hhip^+/+^, n = 23; and HFD-Hhip^+/−^, n = 17); (**c**: ND-Hhip^+/+^, n = 8; ND-Hhip^+/−^, n = 7; HFD-Hhip^+/+^, n = 12; and HFD-Hhip^+/−^, n = 12); (**b**,**d**) plasma circulating insulin level (ng/ml) and its AUC quantifications (total, 0–120 mins; 1^st^ phase, 0–15 mins; 2^nd^ phase, 15–120 mins). (**b**: ND-Hhip^+/+^, n = 11; ND-Hhip^+/−^, n = 7; HFD-Hhip^+/+^, n = 13; and HFD-Hhip^+/−^, n = 13); (**d**: ND-Hhip^+/+^, n = 6; ND-Hhip^+/−^, n = 5; HFD-Hhip^+/+^, n = 11; and HFD-Hhip^+/−^, n = 10). Data shown as mean ± SEM; 1 way-ANOVA followed by Bonferroni’s post hoc test. *p ≤ 0.05; **p ≤ 0.01; ***p ≤ 0.001 vs. ND-Hhip^+/+^; ^ǂ^*p* ≤ 0.05; ^ǂǂ^*p* ≤ 0.01; ^ǂǂǂ^*p* ≤ 0.001 vs. ND-Hhip^+/−^; ^†^*p* ≤ 0.05; ^††^*p* ≤ 0.01; ^†††^*p* ≤ 0.001, HFD-Hhip^+/+^ vs. HFD-Hhip^+/−^; NS, non-significant.

After 8 weeks of HFD, both sexes of 14 week-old HFD-Hhip^+/+^ mice developed a similar pattern of glucose intolerance (male, Fig. 2a; female, Fig. 2c), but hyperinsulinaemia was observed only in male HFD mice (Hhip^+/+^ and Hhip^+/−^) (Fig. 2b), not in female HFD mice (Hhip^+/+^ and Hhip^+/−^) (Fig. 2d), documented by plasma insulin levels measured during intraperitoneal glucose tolerance test (ipGTT). As compared to male HFD-Hhip^+/+^ mice, the impaired ipGTT was ameliorated in male HFD-Hhip^+/−^ mice that had high levels of circulating plasma insulin (ng/ml) in total and/or in two insulin secretion phases (1^st^ phase: 0–15 minutes; 2^nd^ phase: 15–120 mins) during the ipGTT (Fig. 2b). In contrast, those changes did not occur in female HFD mice (Hhip^+/+^ vs. Hhip^+/−^) (Fig. 2d). Therefore, we focused on male mice for the rest of the studies.

**Figure 3:**
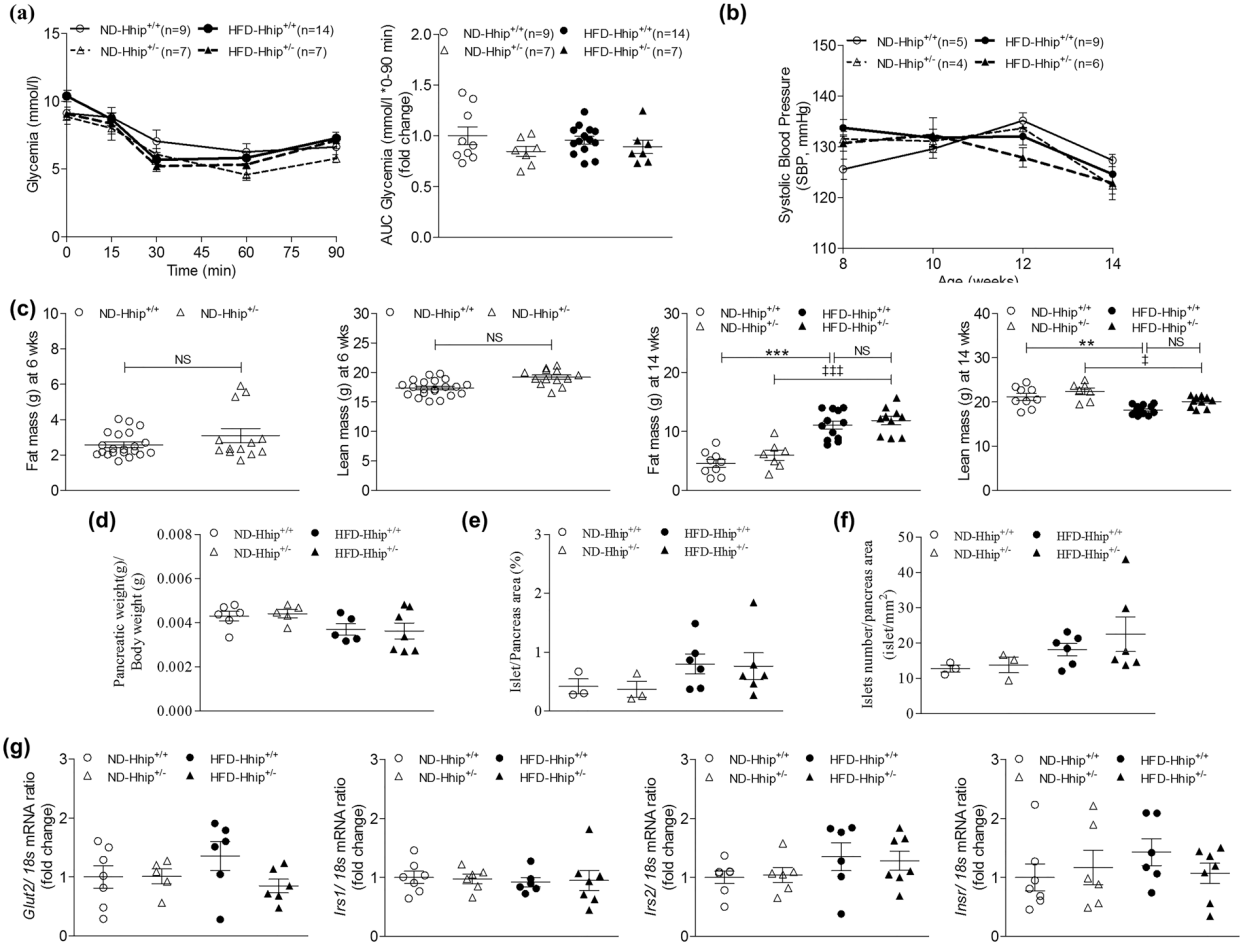
Metabolic and pancreatic parameters in male mice. (**a**) ipIST and ipIST- AUC quantification (0–90 mins) in mice at the age of 14 weeks (ND-Hhip^+/+^, n = 9; ND-Hhip^+/−^, n = 7; HFD-Hhip^+/+^, n = 14; and HFD-Hhip^+/−^, n = 7); (**b**) SBP measurement in mice from the age of 8 to 14 weeks (ND-Hhip^+/+^, n = 5; ND-Hhip^+/−^, n = 4; HFD-Hhip^+/+^, n = 9; and HFD-Hhip^+/−^, n = 6); (**c**) EchoMRI analysis in mice at the age of 6 weeks (ND-Hhip^+/+^, n = 21; ND-Hhip^+/−^, n = 13) and 14 weeks (ND-Hhip^+/+^, n = 9; ND-Hhip^+/−^, n = 7; HFD-Hhip^+/+^, n = 12; and HFD-Hhip^+/−^, n = 10); (**d**–**f**) pancreatic parameters in mice at the age of 14 weeks; (**d**) pancreatic mass (ratio of pancreas weight to BW (ND-Hhip^+/+^, n = 6; ND-Hhip^+/−^, n = 5; HFD-Hhip^+/+^, n = 5; and HFD-Hhip^+/−^, n = 7); (**e**) beta cell mass and (**f**) total islets numbers (ND-Hhip^+/+^, n = 3; ND-Hhip^+/−^, n = 3; HFD-Hhip^+/+^, n = 6; and HFD-Hhip^+/−^, n = 6); and (**g**) qPCR-mRNA expression of *Irs1* (ND-Hhip^+/+^, n = 7; ND-Hhip^+/−^, n = 6; HFD-Hhip^+/+^, n = 5; and HFD-Hhip^+/−^, n = 6*), Irs2* (ND-Hhip^+/+^, n = 7; ND-Hhip^+/−^, n = 6; HFD-Hhip^+/+^, n = 6; and HFD-Hhip^+/−^, n = 7)*, InsR* (ND-Hhip^+/+^, n = 7; ND-Hhip^+/−^, n = 6; HFD-Hhip^+/+^, n = 6; and HFD-Hhip^+/−^, n = 7) and *Glut2* genes (ND-Hhip^+/+^, n = 7; ND-Hhip^+/−^, n = 5; HFD-Hhip^+/+^, n = 6 and HFD-Hhip^+/−^, n = 7) (vs 18 S mRNA ratio), in isolated islets among 4 subgroups of male mice (Hhip^+/+^ vs Hhip^+/−^; ND vs HFD) at 14 week-old. Data shown as mean ± SEM; (**a,b,d**–**f**) 1 way-ANOVA followed by Bonferroni’s post hoc test; (**c**) unpaired student’s *t-*test. **p ≤ 0.01; ***p ≤ 0.001 vs. ND-Hhip^+/+^; ^ǂ^*p* ≤ 0.05; ^ǂǂǂ^*p* ≤ 0.001 vs. ND-Hhip^+/−^; NS, non-significant.

Here, we did not observe any apparent changes in terms of intraperitoneal insulin sensitivity test (ipIST) (Fig. 3a) and longitudinal systolic blood pressure (SBP) measurement (Fig. 3b) among 4 subgroups of male mice (Hhip^+/+^ vs Hhip^+/−^; ND vs HFD). EchoMRI analysis revealed that both male Hhip^+/+^ and Hhip^+/−^ mice had similar fat/lean mass at the age of 6 or 14 weeks in either ND or HFD conditions (Fig. 3c), even though male Hhip^+/−^ mice are generally bigger and heavier through life as compared to male Hhip^+/+^ mice. Next, we analyzed several pancreatic parameters including pancreatic mass (ratio of pancreas weight to BW, Fig. 3d), beta cell mass (Fig. 3e) and *Shh-Glis (Gli 1-Gli3)* mRNA expression (Fig. S1), as well as total islets numbers (Fig. 3f) in the isolated islets among 4 subgroups of male mice (Hhip^+/+^ vs Hhip^+/−^; ND vs HFD) and we did not find any apparent changes, as well as no changes in mRNA expression levels of *Irs 1, Irs 2, InsR* and *Glu2* genes in those isolated islets (Fig. 3g).

### Pancreatic Hhip expression and glucose stimulated insulin secretion (GSIS)

**Figure 4:**
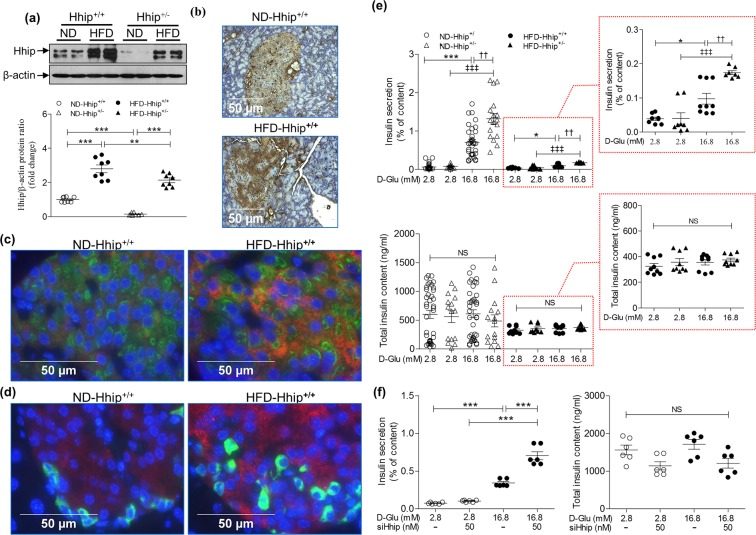
Pancreatic Hhip expression (**a–d**) and GSIS (**e,f**). (**a–d**) Hhip expression in the islets of male Hhip mice (Hhip^+/+^ vs. Hhip^+/+^; ND vs HFD) at 14 week-old; (**a**) Western blot; (**b**) Hhip-IHC staining (scale bar, 50 μm); (**c**) co-localization of IF-Hhip (red), IF-insulin (green) and DAPI (blue); (**d**) co-localization of IF-Hhip (red), IF-glucagon (green) and DAPI (blue); (**e**) GSIS in isolated islets from male Hhip^+/+^ vs Hhip^+/−^ mice at 14 week-old under ND and HFD conditions (insulin secretion, % of total insulin content (ng/ml) measured in isolated islets cultured at 2.8 vs. 16.8 mM D-glucose medium). *p ≤ 0.05; ***p ≤ 0.001 vs. ND-Hhip^+/+^; ^###^*p* ≤ 0.001 vs. ND-Hhip^+/−^; ^††^*p* ≤ 0.01, HFD-Hhip^+/+^ vs. HFD-Hhip^+/−^; NS, non-significant. (**f**) siRNA-Hhip (50 nM) effect on GSIS in INS-1 832/13 cells (insulin secretion, % of total insulin content (ng/ml) measured in INS-1 832/13 cells cultured at 2.8 or 16.8 mM D-glucose medium). ***p ≤ 0.001 vs. INS-1 832/13 cells cultured in 2.8 mM D-glucose medium). NS, non-significant. Three to four separate experiments (*ex vivo* and *in vitro*); Data shown as mean ± SEM; 1 way-ANOVA followed by Bonferroni’s post hoc test.

Although Hhip is abundantly expressed in the developing pancreas^9^ and in vascular endothelial cells^19^, less is known about its basal expression level in mature islet cells such as alpha and beta cells, and also, whether HFD could alter its expression profile in these islet cells and subsequently impact on insulin secretion/action. As compared to ND, HFD stimulated Hhip protein expression in freshly isolated islets of both Hhip^+/+^ and Hhip^+/−^ mice, but, less in HFD- Hhip^+/−^ mice vs HFD- Hhip^+/+^ mice by western blot (Fig. 4a). HFD increased Hhip-IHC expression in the islets of Hhip^+/+^ mice (Fig. 4b), predominantly evident in beta cells, not alpha cells (co-localized with insulin (Fig. 4c) and glucagon (Fig. 4d)). In addition, *ex vivo* studies revealed that the blunted GSIS in HFD mice reduced insulin secretion, ~7-fold less than in ND animals (Fig. 4e). Islets of Hhip heterozygous (Hhip^+/−^) mice had enhanced GSIS (ND, ~1.8 fold increase; HFD, ~1.6 fold increase) as compared to Hhip^+/+^ mice under ND and HFD conditions (Fig. 4e). In INS-1 832/13 cells *in vitro*, siRNA-Hhip (50 nM) further enhanced the stimulatory effects of 16.8 mM glucose on GSIS (Fig. 4f).

### Islet integrity and beta cell morphology

**Figure 5:**
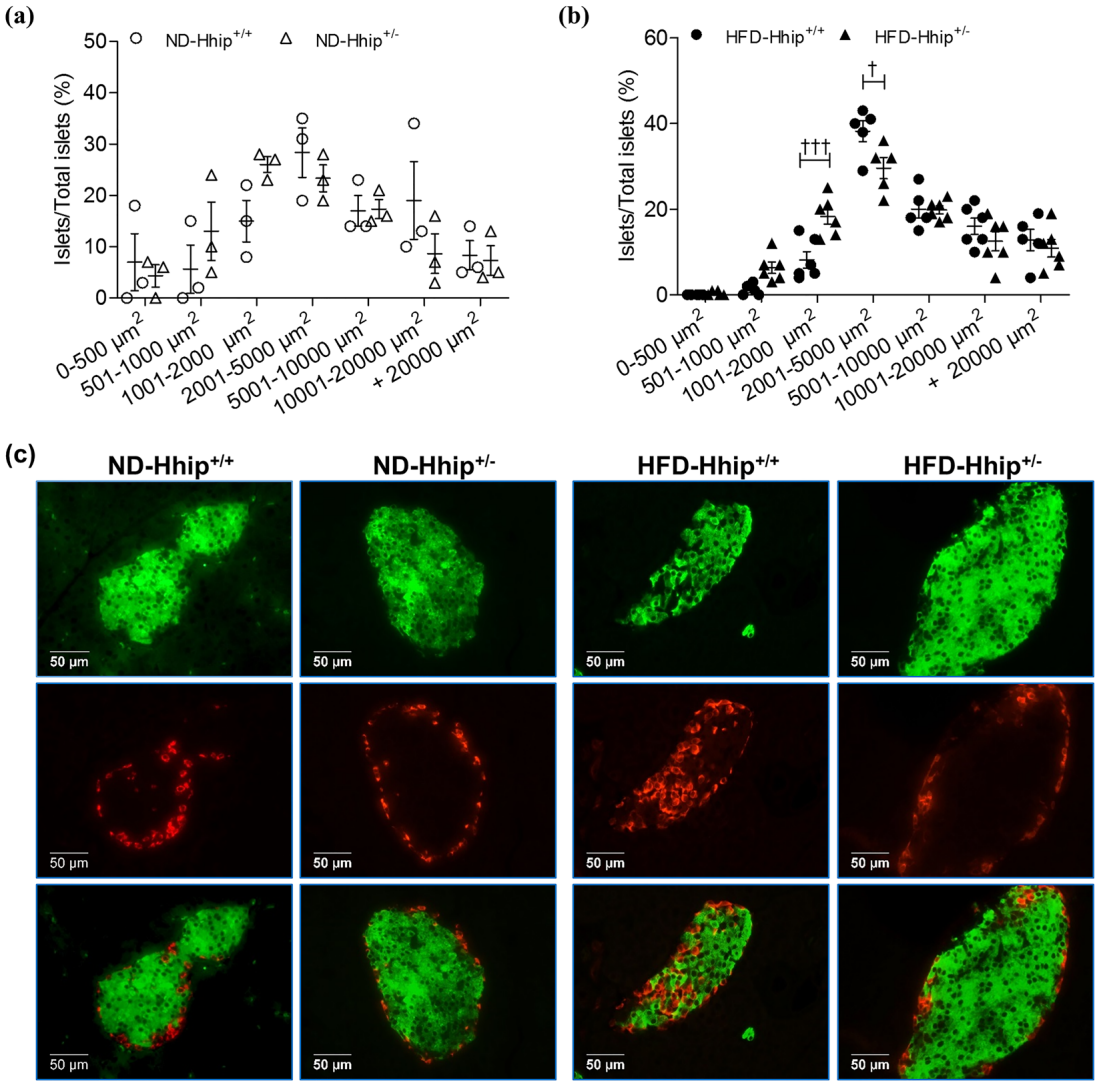
Islet analysis of male mice at 14 week-old (Hhip^+/+^ vs Hhip^+/−^; ND vs HFD*)*. (**a**,**b**) Distribution profile of islet size [**a**, ND (ND-Hhip^+/+^, n = 3; ND-Hhip^+/−^, n = 3); (**b)**, HFD (HFD-Hhip^+/+^, n = 6; HFD-Hhip^+/−^, n = 6)]. Data shown as mean ± SEM; 2 way-ANOVA followed by Bonferroni’s post hoc test. ^†^*p* ≤ 0.05; ^†††^*p* ≤ 0.001, HFD-Hhip^+/+^ vs. HFD-Hhip^+/−^; (**c**) co-localization of IF-insulin (green) and –glucagon (red) staining (scale bar, 50 μm).

**Figure 6:**
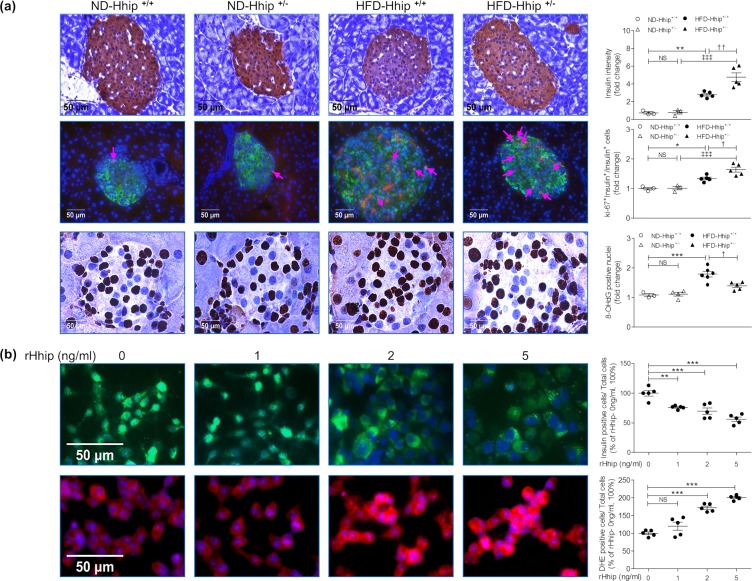
IHC/IF staining *in vivo* (**a**) and *in vitro* (**b**). (**a**) Insulin-IHC (ND-Hhip^+/+^, n = 3; ND-Hhip^+/−^, n = 3; HFD-Hhip^+/+^, n = 5; and HFD-Hhip^+/−^, n = 5); IF-Ki67 staining (Ki67, red; insulin, green; DAPI, blue; Ki-67 positive nuclei, pink arrows) (ND-Hhip^+/+^, n = 3; ND-Hhip^+/−^, n = 3; HFD-Hhip^+/+^, n = 5; and HFD-Hhip^+/−^, n = 5) and 8-OHdG-IHC staining (ND-Hhip^+/+^, n = 4; ND-Hhip^+/−^, n = 3; HFD-Hhip^+/+^, n = 6; and HFD-Hhip^+/−^, n = 5) in the islets among 4 subgroups of male mice (Hhip^+/+^ vs Hhip^+/−^; ND vs HFD) at 14 week-old (scale bar, 50 μm). Semi-quantification of staining; Data shown as mean ± SEM; 1 way-ANOVA followed by Bonferroni’s post hoc test. *p ≤ 0.05; ***p ≤ 0.001 vs. ND-Hhip^+/+^; ^ǂǂǂ^*p* ≤ 0.001 vs. ND-Hhip^+/−^; ^†^*p* ≤ 0.05 HFD-Hhip^+/+^ vs. HFD-Hhip^+/−^; NS, non-significant. (**b**) IF-insulin staining (insulin, green; DAPI, blue) and DHE staining (DHE, red; DAPI, blue) (scale bar, 50 µm); Semi-quantification; Four separate experiments; Data shown as mean ± SEM; 1 way-ANOVA followed by Bonferroni’s post hoc test. **p ≤ 0.01; ***p ≤ 0.001; NS, non-significant vs INS-1 832/13 cells cultured in medium without rHhip (0 ng/ml) (100%).

Next, we analyzed the frequency distribution profile of islet size by measuring islet surface areas from 0–500 µm^2^ to over 20000 µm^2^ in animals under both ND (Fig. 5a) and HFD (Fig. 5b) conditions. It appeared that ND-Hhip^+/−^ and ND-Hhip^+/+^ mice displayed a similar islet-size distribution pattern (Fig. 5a). However, HFD-Hhip^+/−^ had relatively more smaller-sized islets (<2000 µm^2^) as compared to HFD-Hhip^+/+^ mice, while HFD-Hhip^+/+^ mice had an increase in larger-sized islets (>2000 µm^2^) (Fig. 5b). Notably, morphological features including disoriented islet architecture with an invasion of α-cells into the central core of beta cells (Fig. 5c, insulin-IF staining and glucagon-IF staining) were observed in the pancreases of HFD-Hhip^+/+^ mice. More specifically, reduced insulin content (Fig. 6a, insulin-IHC staining) and decreased beta cell proliferation (Fig. 6a, Ki-67-IF staining) occurred significantly more often in the pancreases of HFD-Hhip^+/+^ mice. In contrast, HFD-Hhip^+/−^ mice had a preponderance of smaller-sized islets (<2000 µm^2^) (Fig. 5b), in which islet integrity (Fig. 5c) and insulin content (Figs 5c and 6a) were preserved and protected, while increased beta cell proliferation (Fig. 6a) was detected as well.

### Oxidative stress

8-hydroxy-2-deoxyguanosine (8-OHdG) staining (Fig. 6a) revealed that oxidative stress was markedly elevated in the islets of HFD-Hhip^+/+^ mice vs. HFD-Hhip^+/−^ mice, while no differences were observed between ND-Hhip^+/+^ mice and ND-Hhip^+/−^ mice. *In vitro*, recombinant Hhip (rHhip) dose-dependently decreased and increased the numbers of insulin-positive cells (Fig. 6b) and dihydroethidium (DHE)-positive cells (Fig. 6b), respectively.

**Figure 7:**
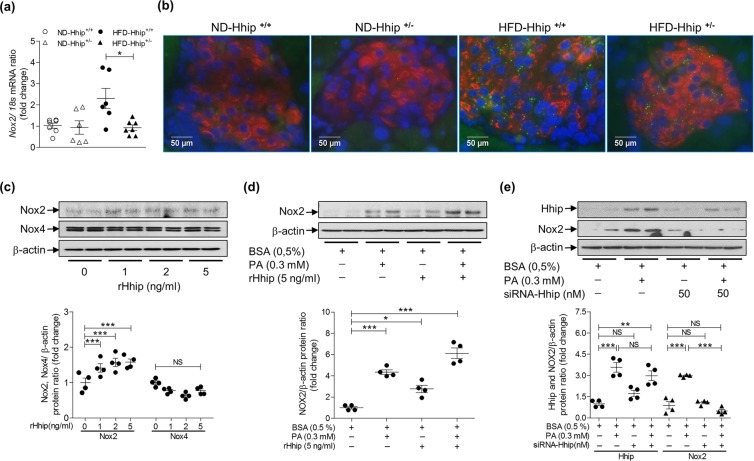
*Nox2* gene expression *in vivo* (**a,b**) and *in vitro* (**c–f**). (**a**) qPCR –*Nox2* mRNA expression (ND-Hhip^+/+^, n = 7; ND-Hhip^+/−^, n = 6; HFD-Hhip^+/+^, n = 6; and HFD-Hhip^+/−^, n = 7); Data shown as mean ± SEM; 1 way-ANOVA followed by Bonferroni’s post hoc test. *p ≤ 0.05, HFD-Hhip^+/+^ vs. HFD-Hhip^+/−^ male mice at 14 week-old; (**b**) IF-Nox2 staining (Nox2, green; insulin, red; DAPI, blue) in the islets among 4 subgroups of male mice (Hhip^+/+^ vs Hhip^+/−^; ND vs HFD) at 14 week-old (scale bar, 50 μm). (**c**) WB (Nox2 and Nox4 protein expression). The cells were treated by rHhip (0–5 ng/ml). (**d,e**) WB (Nox2 protein expression). The cells were treated by PA (0.3 mM) with rHhip (5 ng/ml) (**d**) or siRNA-Hhip (50 nM) (**e**). Three to four separate experiments; Data shown as mean ± SEM; 1 way-ANOVA followed by Bonferroni’s post hoc test; *p ≤ 0.05; ***p ≤ 0.001; NS, non-significant vs INS-1 832/13 cells cultured in medium with 0.5% BSA (100%).

Then, we analyzed NADPH oxidase (*Nox 1*, *2* and *4* genes) mRNA expression by qPCR in isolated mouse islets and found that only *Nox 2* mRNA was significantly increased in the islets of HFD-Hhip^+/+^ mice vs. HFD-Hhip^+/−^ mice (Fig. 7a), while *Nox 1* and *Nox* 4 mRNA were barely detectable in those islets (data not shown). Co-IF staining (Nox2 and insulin) further confirmed that the elevated Nox2-IF expression was far greater in the beta cells of HFD-Hhip^+/+^ mice vs. HFD-Hhip^+/−^ mice (Fig. 7b). In INS-1 832/13 cells, we could detect both *Nox 2* and *Nox 4* mRNA expression, but not *Nox 1* mRNA expression. As shown, rHhip dose-dependently increased *Nox2* gene expression (mRNA, Fig. S2a; protein, Fig. 7c) and elevated NADPH activity (Fig. S2b), while *Nox4* gene expression remained unchanged (mRNA, Fig. S2a; protein, Fig. 7c). In addition, 0.3 mM BSA-sodium palmitate (PA) stimulated *Nox2* mRNA expression without affecting *Nox4* mRNA expression (Fig. S2c). The immunoblotting data revealed that 0.3 mM BSA-PA stimulated both Hhip and Nox2 protein expression, which was increased by rHhip (Fig. 7d) or abolished by siRNA-Hhip (Fig. 7e).

## Discussion

In the present study, we demonstrated that HFD stimulates *Hhip* gene expression in beta cells, resulting in disruption of islet integrity and decreased GSIS. Mechanistically, Hhip promotes oxidative stress in beta cells via the *Nox 2* gene.

As expected, 8 weeks of HFD increased body weight over time in both sexes of HFD-Hhip^+/+^ and HFD-Hhip^+/−^ mice. Despite the fact that female sex has been reported to be protective against HFD-induced metabolic disorders^23–25^, both sexes of HFD-Hhip^+/+^ mice displayed a similar ipGTT pattern in our study, and the impaired glucose intolerance was improved only in male HFD-Hhip^+/−^ mice, but not in female HFD-Hhip^+/−^ mice. To date, a few publications have explicitly looked at the role of sex in the response to HFD in mice^23,24,26–28^. Notably, HFD impact on metabolic phenotypes largely depends on sexually dimorphic variables, but strains, animal ages and environmental factors may be confounders^23,24,26–28^. Here, the sexual dimorphism might be explained by (1) the mixed genetic background in our JAX® Hhip mouse line. For instance, by using gold-standard methodologies, Fergusson *et al*. reported that C57BL/6J and C57BL/6N mice in response to glucose challenge to secrete insulin are completely different^29^. (2) the impact of body weight. As an example, Ingvorsen *et al*. studied the impact of diet and sex on 41 metabolic related variables from 1319 C57BL/6N mice (ND, N = 586 vs. HFD, N = 733) and found that for the majority of variables (79%), body weight on HFD-induced metabolic changes is a significant source of sexually dimorphic variation^28^. Nevertheless, seeing the differences between sexes, we then focused on male animals to elucidate the relevant mechanisms.

Glucose uptake and insulin secretion are the best known mechanisms that impact on systemic glucose tolerance. Relevant to the pancreas, insulin has been shown to be involved in glucose uptake from circulation into peripheral insulin-responsive tissues through glucose transporters, such as Glut2 in pancreatic islets^30^. Under basal conditions, Glut2 level is very low (sequestered internally), but in the presence of insulin (with the appropriate stimulus such as HFD), the expression of Glut2 might be altered to maintain glucose homeostasis^30–32^. Here, we did not observe any apparent changes of *Glut2* mRNA in pancreatic islets, hinting that insulin-mediated glucose uptake in pancreatic islets might be normal. In line with others^33–35^, mouse islets express low levels of classical Shh signaling, underscoring the independence of Hhip action. Our data also revealed that the glucose response to exogenous insulin challenge (ipIST) and insulin signaling (*Irs 1, Irs 2* and *InsR* mRNA) remained at similar levels among the 4 subgroups of mice fed either ND or HFD; and there were not any apparent changes in pancreatic mass, beta cell mass and total islet numbers in those experimental animals. Notably, depending on the composition and timing of diet, beta cells respond to HFD feeding in mice with two actions–insulin compensation (which results in hyperinsulinemia) and/or insulin resistance^24,36–41^. For example, within a 10-week period of HFD intake, beta cells are prone to secrete more insulin (hyperinsulinemia) to compensate the increasing obesity and glucose intolerance^24,38^, while long-term HFD consumption (more than 10 weeks) eventually culminates in beta cell failure and the change of those parameters is considered a major adaptation to insulin resistance^36–39,41^. Being neither insulin insensitivity nor insulin resistance, the current model with 8-week HFD might only induce beta cell proliferation as reported^24,38^.

Thus, we speculate that the impaired ipGTT observed in HFD-Hhip^+/+^ mice might be as a result of pancreatic beta cell dysfunction on GSIS. Indeed, we here found that Hhip expression was significantly increased in the islets of HFD-Hhip^+/+^ mice (>HFD- Hhip^+/−^ mice), mainly co-localized in beta cells and none in alpha cells, pin-pointing beta cells as a main target of HFD-induced Hhip expression. The islets isolated from Hhip^+/−^ mice under both ND and HFD diet had enhanced GSIS responses as compared to those from Hhip^+/+^ mice. Similarly, siRNA-Hhip in INS-1 832/13 cells resulted in an increase of GSIS in response to glucose challenge from 2.8 mM to 16.8 mM. Together, these data suggest that Hhip deficiency might directly preserve the capacity and ability of GSIS in insulin production and lower *Hhip* gene expression could enhance GSIS, and that might also explain the phenotype of HFD- Hhip^+/−^ mice showing an improved ipGTT with higher circulating plasma insulin levels in two insulin secretion phases. We are unable to capture the obvious expected 1st phase of insulin secretion (*data not shown*), that result might be due to the mixed genetic background of our mice. As noted by others^42–44^, glucose metabolism on insulin secretory function indeed varies in a strain-dependent manner.

Islet size, insulin content and the amount of insulin released are known to be correlated in proportional population existing in animals and humans, evidenced by small islets that are comprised of more beta cells with higher insulin content than large islets^45,46^. When there is increased insulin demand as in diabetes and obesity, islet cell composition and size distribution pattern appear to be altered, and some islets exhibit abnormal architecture with intermingled alpha and beta cells, particularly large islets and distribution with a disproportionate number of larger size islets populations^45,47^. As noted, HFD-Hhip^+/+^ mice phenotypically had larger-sized islets with alpha cell invasion, lower insulin content and less beta cell proliferation. In contrast, Hhip deficiency protected islet integrity, in response to HFD challenge over time; and there were increased numbers smaller islets and beta cell proliferation in HFD-Hhip^+/−^ mice, thereby improving GSIS.

Mechanistically, evidence indicates that under oxidative stress, *Hhip* gene expression may redirect cells toward apoptosis, fibrosis, angiogenesis and/or tumorigenesis^19,48–50^. Previously, we demonstrated that Hhip could directly elevate ROS generation; interact with NOX isoform(s) (i.e., Nox 4 in the kidney), subsequently activating TGFβ1-signaling to result in renal cell fibrosis/apoptosis, irrespective of diabetes^50,51^. Here, oxidative stress in the islets and beta cells was markedly elevated in HFD-Hhip^+/+^ mice vs. HFD-Hhip^+/−^ mice. *In vitro*, rHhip dose-dependently decreased insulin-positive beta cells number; increased the number of DHE-positive cells and NADPH activity. Together, these data suggest that HFD-increased *Hhip* gene expression might elevate oxidative stress in beta cells, thereby impairing GSIS on insulin secretion.

In the pancreas, the NOX family represents one potential source of ROS in insulin-secreting cells^22^. Among NOX isoforms, we detected *Nox2* gene expression (islets and INS-1 832/13 cells); *Nox4* (INS-1 832/13 cells); but not *Nox1* (neither islets nor INS-1 832/13 cells), in line with others^21,22^. Also, only *Nox2* (but not *Nox4*) gene expression was elevated in the islets of HFD-Hhip^+/+^ mice, and that elevated Nox2 expression was ameliorated in the islets of HFD-Hhip^+/−^ mice. Currently, it is still unclear whether Nox2 has an impact on GSIS or not, since both positive^21^ and negative^52^ results have been reported in Nox2 KO mice (C57BL/6) (Jackson Laboratory, Bar Harbor, ME) as well. Nevertheless, we further validated Hhip impact on *Nox2* gene expression *in vitro*. We found that rHhip directly stimulated Nox2 expression (mRNA and protein) in a dose-dependent manner; 0.3 mM BSA-PA stimulated both Hhip and Nox2 protein expression, that could be either aggravated or abolished by rHhip or siRNA-Hhip, respectively. Taken together, not only did we confirm the notion of Nox2 acting as the predominant isoform and therefore Nox2 elevates ROS production to antagonize GSIS in the regulation of insulin secretion^21^, but we also established that a lower *Hhip* gene expression might protect islet integrity against HFD-mediated beta cell dysfunction; improve GSIS on maintaining sufficient levels of insulin secretion via ameliorating ROS-*Nox2* gene expression. Clearly, beta cell-specific gain- and/or loss-of-Hhip function/expression models would be merited to circumvent the potential pitfall of the current whole body Hhip-deficient model in the future.

In conclusion, pancreatic *Hhip* gene regulates insulin secretion by altering islet integrity and promoting *Nox2* gene expression in beta cells in response to HFD-mediated beta cell dysfunction. As a perspective, exploring a method to decrease/lower Hhip expression may provide a new therapeutic strategy in the diagnosis, prevention and treatment of T2D.

## Materials and Methods

### Animal models

Heterozygous Hhip (Hhip^+/−^) mice and control littermates (Hhip^+/+^) (Jackson Laboratories, Hhip^*tm1Amc*^/J; mixed background of C57BL/6, Swiss-Webster, 129) were used (N.B., Adult Hhip^+/–^ mice are phenotypically indistinguishable from control littermates (Hhip^+/+^), whereas Hhip^−/−^ die after birth due to lung defects; thus, Hhip^+/−^ mice were used in the current study^9,12^). In brief, both sexes of Hhip mice (Hhip^+/−^ vs. Hhip^+/+^) at the age of 6 weeks were fed with normal chow (ND) (18% protein with 6.2% fat, calories from protein 24%, fat 18% and carbohydrate 58%) (Harlan Teklad, Montreal, Canada) or HFD (20.5% protein with 36% fat, calories from protein 14%, fat 60% and carbohydrate 26%) (Bio-Serv, Flemington, NJ) until 14 weeks of age, as reported previously^53^. After euthanasia (75 mg/kg sodium pentobarbital i.p.), pancreata were rapidly processed and harvested for either islets isolation or fixation for immunohistochemistry (IHC) and/or immunofluorescence (IF).

All animal protocols were carried out in strict accordance with the recommendations in the NIH Guide for the Care and Use of Laboratory Animals and followed the Principles of Laboratory Animal Care [National Institutes of Health (NIH) publication no. 85–23, revised 1985: http://grants1.nih.gov/grants/olaw/references/phspol.htm]. Animal care and experimental procedures were approved by the Animal Care Committee from the Centre de recherche du centre hospitalier de l’Université de Montréal (CRCHUM). Animals were housed in ventilated cages in SPF conditions under a 12 hours light-dark cycle with free access to chow and water at the animal facility of the CRCHUM. Breeding was carried out in pairs (Hhip^+/+^ with Hhip^+/−^ mating) under the same housing conditions.

### Biological parameters measurement

Body weight (BW, g) and energy intake (kCal/week) were monitored weekly through the experimental course. With the aid of the Rodent Cardiovascular Phenotyping Core Facility (CRCHUM), fat and lean mass as a percentage of body weight for mice were assessed using an EchoMRI-700 (EchoMRI™, Houston, TX); and plasma insulin was measured by mouse ultrasensitive insulin ELISA jumbo kit (Alpco Diagnostics, Salem, NH). Longitudinal systolic blood pressure (SBP) (from the age of 8 until 14 weeks following one week of pre-training) was monitored by the tail-cuff method with a BP-2000 Blood Pressure Analysis System (Visitech Systems Inc., Apex, NC), as reported elsewhere^50,53^. Intraperitoneal glucose tolerance test (ipGTT) and insulin sensitivity test (ipIST) were performed according to a standard protocol, with 6 and 4 hours fasting periods before sacrifice at the age of 14 weeks, respectively^53^. Blood glucose was quantified with an Accu-Chek Performa glucose meter (Roche Diagnostics, Laval, QC, Canada)^50,53^.

### Islet isolation and glucose stimulated insulin secretion

Islet isolation was performed following a previously reported protocol^54^. In brief, mouse islets were isolated by collagenase P digestion and purified on a bilayer HBSS/Histopaque 1091 and 1077 (Sigma-Aldrich Canada, Oakville, ON, Canada) gradient centrifugation. Freshly isolated islets were used for Western Blot (WB) and qPCR analysis. For glucose-stimulated insulin secretion (GSIS) experiments, isolated islets were kept in culture at 37 °C in 2.8 mM glucose RPMI complete medium supplemented with 10% fetal bovine serum (FBS) for overnight recovery. GSIS and insulin content in both islets and culture media were assessed in 1-hour static incubation in Krebs-Ringer bicarbonate buffer containing 10 mM HEPES (pH 7.4, KRBH), 0.5% defatted BSA (d-BSA) at 2.8 mM glucose and 16.8 mM glucose, respectively (CRCHUM Cellular Physiology Core Facility).

### Islets numbers, size and beta cell mass

With the aid of the CRCHUM Cellular Physiology Core Facility, we measured several islet parameters including total islet number, size and beta cell mass, as reported^29,55^. In brief, whole pancreata were removed, weighed, fixed and embedded in paraffin blocks. Longitudinal pancreatic cross sections (5 μm thick) were collected at 30 μm intervals. Insulin-IHC (anti-guinea pig insulin antibody, Agilent-DAKO, Santa Clara, CA) with counterstaining with hematoxylin was performed according to a standard protocol. At least 4–6 slides from each pancreas were processed for islet and beta cell mass measurements. First, slides were scanned at 20X magnification by using an Aperio ScanScope model CS slide scanner (Leica Biosystems Inc., Concord, ON, Canada) to assess islet/beta cell area and whole pancreas area via the Aperio Pixel count algorithm v9 (ImageScope v12.3.2.5030, Leica Biosystems Inc.), followed by calculation of the ratio of beta cell area to whole pancreas area. Then, beta cell mass was calculated by multiplying the ratio of beta cell area to whole pancreas area with whole pancreatic mass (mg) measured before fixation. Finally, morphometric measurements were performed by identifying manually regions of interest (ROIs) around insulin-IHC positive islets. The surface of all islets from at least 4 sections (>400 islets) were calculated for each ROI (ImageScope) and used to generate the size frequency distribution (surface) profile.

### Cell culture

We employed a pancreatic beta cell line, rat INS-1 832/13^56,57^ kindly provided by Dr. Marc Prentki (CRCHUM, Montreal, QC, Canada) for our *in vitro* study. In brief, INS-1 832/13 cells (passage 51–53) have robust glucose responsiveness over the physiological range of glucose concentrations (i.e., 2.8–16.8 mM glucose) and were cultured in RPMI 1640 medium supplemented with 10% FBS, 10 mM HEPES, 2 mM L-glutamine, 1 mM sodium pyruvate, and 50 µM β-mercaptoethanol.

### Oxidative stress and real-time quantitative PCR (qPCR)

Oxidative stress was determined by 8-hydroxy-2-deoxyguanosine (8-OHdG) staining (a biomarker for oxidative stress or a marker for reactive oxygen species (ROS) damage)^58^; dihydroethidium (DHE) staining (a cell-permeable fluorescent dye, redox indicator), NADPH oxidases (*Nox 1, 2* and *4*) gene expression and NADPH activity^50,51,59,60^. qPCR (Fast SYBR green master mix kit and 7500 Fast real-time PCR system; Applied Biosystems, Foster City, CA, USA) was performed as reported previously^50,51^. Primer sequences for qPCR were listed in Table 1.

**Table 1:** Primers sequences.

Gene	Primer sequences	Reference Sequence
18S ribosomal RNA	S: AGTCCCTGCCCTTTGTACACA AS: CGATCCGAGGGCCTCACTA	NR_003278.3
β-actin (Rat)	S: ATCGGCAATGAGCGGTTCC AS: AGCACTGTGTTGGCATAGAGG	NM_031144.3
Glut2	S: CACACCAGCATACACAACACCAG AS: GGACACAGACAGAGACCAGAGC	NM_031197.2
Insr	S: CATGTGCAGGAATGGCTTGTT AS: TTCTGCGTTTTCTGCAGTGCTA	NM_010568.3
Irs1	S: AATCCTCAGGAGTTCATTGACTGAA AS: TTCCGGTGTCACAGTGCTTTC	NM_010570.4
Irs2	S: GGCCCGAACCTCAATAACAA AS: CCGCGCAACACGAAAAAG	NM_001081212.2
Nox2 (Mouse)	S: TGTGGTTGGGGCTGAATGTC AS: CTGAGAAAGGAGAGCAGATTTCG	NM_007807.5
Nox2 (Rat)	S: CCCTTTGGTACAGCCAGTGAAGAT AS: CAATCCCAGCTCCCACTAACATCA	NM_023965.1
Nox4 (Mouse)	S: GAAGGGGTTAAACACCTCTGC AS: ATGCTCTGCTTAAACACAATCCT	NM_015760.5
Nox4 (Rat)	S: TGGCCAACGAAGGGGTTAAA AS: GATCAGGCTGCAGTTGAGGT	NM_053524.1

### Reagents and chemicals

Antibodies (method with dilution) used included anti-Hhip (5D11) (WB, 1:2000; IF/IHC, 1:300), glucagon (IF, 1:200), β-actin (WB, 1:100000) from Sigma-Aldrich Canada (Oakville, ON, Canada); anti-Ki 67 (IF, 1: 50) from BD Bioscience (San Jose, CA, USA); anti-insulin (H-86) (IF/IHC, 1:500) and gp91-phox antibody (K-15) (IF, 1:200) from Santa Cruz Biotechnology (Santa Cruz, CA, USA); anti-mouse 8-OHdG (IHC, 1: 50) from Abcam (Toronto, ON, Canada). Mouse anti-gp91phox-Cter for WB (1:10000) was obtained from Dr. Nathalie Grandvaux (CRCHUM) as reported elsewhere^61^. Chemical reagents included small interfering RNA (siRNA) of Hhip from Santa Cruz Biotechnology (Santa Cruz, CA, USA), which pools three target-specific 19–25 nucleotide sequences (Fig. S2e); recombinant Hhip (rHhip) from R&D Systems, Inc. (Burlington, ON, Canada); BSA (fatty acid free) and sodium palmitate (PA) were procured from Sigma-Aldrich Canada. The preparation of 0.3 mM BSA-PA (vs. the control, 0.5%BSA) was done as reported elsewhere^53,62^. Semi-quantitation of relative staining values was performed by NIH Image J software (Bethesda, MD). The images (N = 8~15 per animal) were analyzed and quantitated in a randomized and blinded fashion.

### Statistical analysis

For animal studies, groups of 8 to 23 mice were studied (N.B. The precise number of animals used for each specific experiment is either labeled in the figures or the individual data points are shown on a column scatter graph). *Ex vivo* and *in vitro*, three to four separate experiments were performed for each protocol. All values represent mean ± SEM. Statistical significance between the experimental groups was analyzed by Student’s t-test, 1-way ANOVA and/or 2-way ANOVA (islet size frequency distribution) followed by the Bonferroni test using Prism 5.0 software (GraphPad, San Diego, CA). A probability level of p ≤ 0.05 was considered to be statistically significant^50,53^.

## Supplementary information

Supplemental Figures (S1-S3)
