# Supplementary material for: Hedgehog Interacting Protein (Hhip) Regulates Insulin Secretion in Mice Fed High Fat Diets
Source: Sci Rep. 2019 Aug 1;9:11183. doi: 10.1038/s41598-019-47633-3 (PMC6673691; doi:10.1038/s41598-019-47633-3)
Supplement: Supplementary file 1 — Supplemental Figures (S1-S3) [file 41598_2019_47633_MOESM1_ESM.pdf]

Hedgehog Interacting Protein (Hhip) Regulates Insulin Secretion in Mice Fed High Fat Diets

Henry Nchienzia[1], Min-Chun Liao[1], Xin-Ping Zhao[1], Shiao-Ying Chang[1], Chao-Sheng Lo[1], Isabelle Chenier [1], Julie R. Ingelfinger [2], John S.D. Chan[1] and Shao-Ling Zhang [1§]

[1]Université de Montréal
Centre de recherche du Centre hospitalier de l'Université de Montréal (CRCHUM)
Tour Viger, 900 rue Saint-Denis, Montréal, QC, Canada H2X 0A9

[2] Harvard Medical School
Pediatric Nephrology Unit
Massachusetts General Hospital
55 Fruit Street, Boston, MA 02114-3117, USA

[§] To whom correspondence should be addressed: Shao-Ling Zhang, Ph.D.

Tel: (514) 890-8000 ext. 15633
Fax: (514) 412-7204
Email: shao.ling.zhang@umontreal.ca

Short Title: Hedgehog interacting protein (Hhip) gene and pancreatic beta cell dysfunction

Key words: High Fat Diet, Hhip Gene Expression, Pancreatic beta Cell Dysfunction

Word Count: Abstract 199; Main Text 3000; Method 1218

(a) Mouse Islets-qPCR

Mouse Shh (NM_009170.3)	
m-Shh-S	AAAGCTGACCCCTTTAGCCTA
m-Shh-AS	TTCGGAGTTTCTTGTGATCTTCC

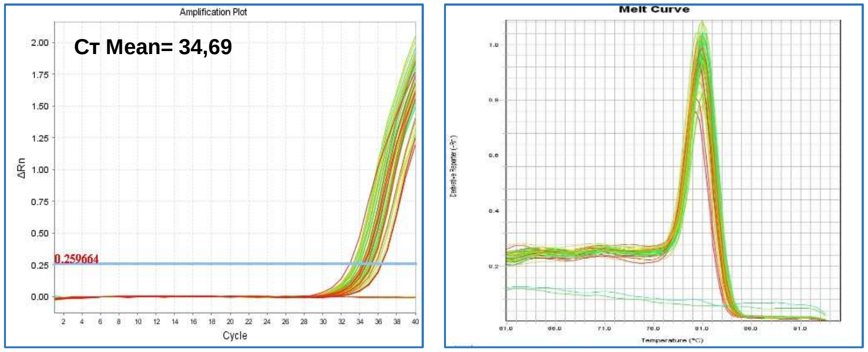

Mouse Gli1 (NM_010296.2)	
m-Gli1-S	GGAAGTCCTATTCACGCCTTGA
m-Gli1-AS	CAACCTTCTTGCTCACACATGTAA

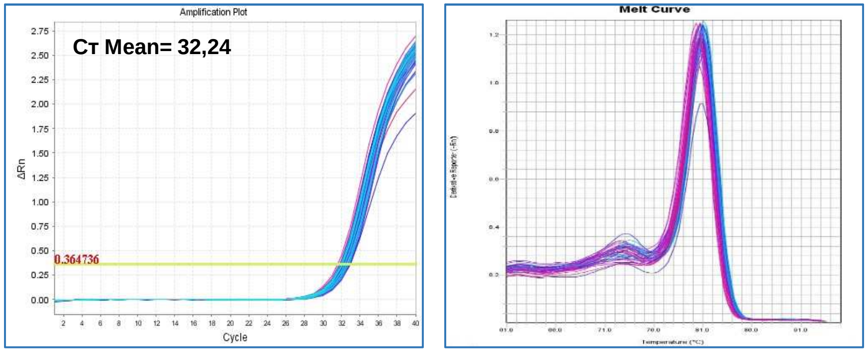

Mouse Gli2 (NM_001081125.1)	
m-Gli2-S	CATGGTATCCCTAGCTCCTC
m-Gli2-AS	GATGGCATCAAAGTCAATCT

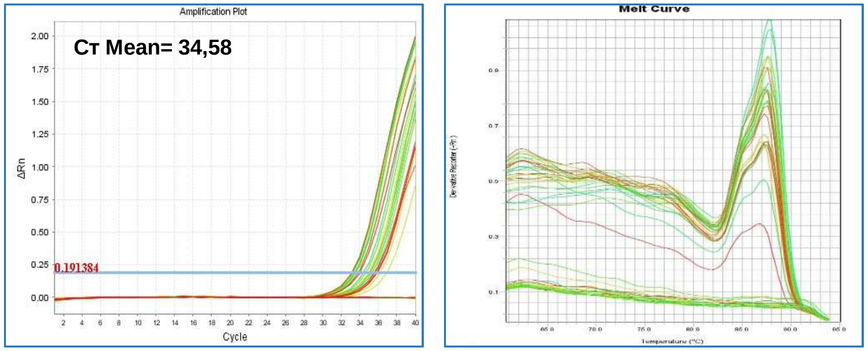

Mouse Gli3 (NM_008130.2)	
m-Gli3-S	CATGAACAGCCCTTTAAGAC
m-Gli3-AS	TGATATGTGAGGTAGCACCA

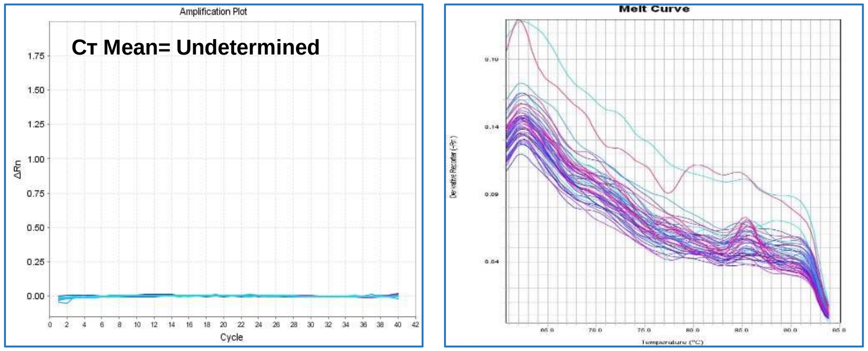

(b) qPCR Protocol

qPCR cycling protocol		
Activation	Denature	Anneal/ extend
2 min @ 95°C	40 cycles, 3 sec @ 95°C	40 cycles, 30 sec @ 60°C

Fig. S1. Shh-Glis mRNA expression in male mouse islets (Hhip $^{+/+}$ vs Hhip $^{+/-}$; ND vs HFD) at 14 week-old. (a) qPCR data (CT and melting curves; Primers Sequences); (b) qPCR cycling protocol

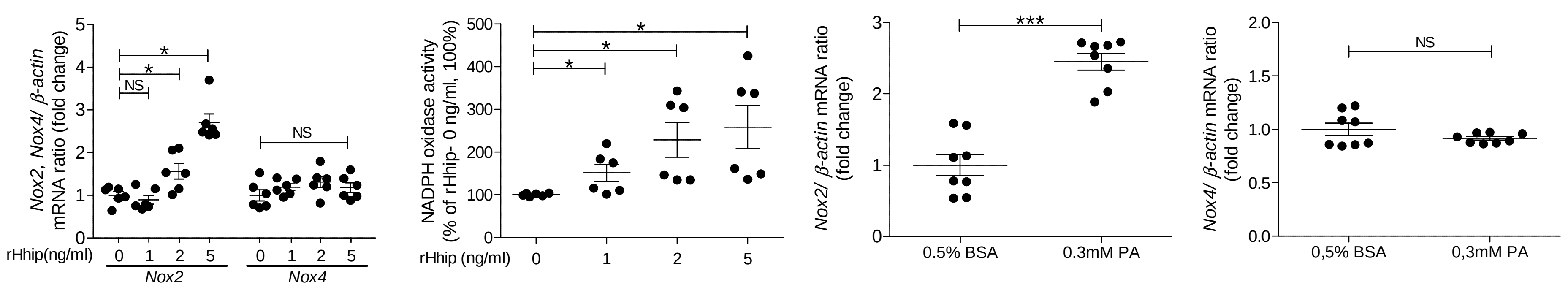

(e)

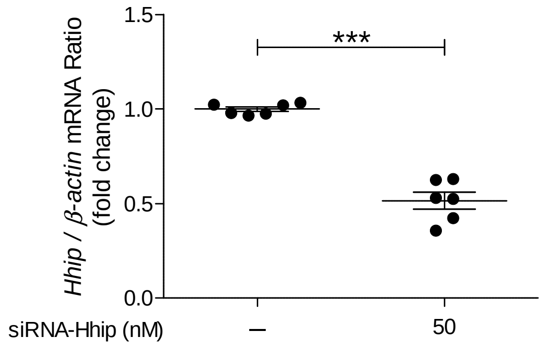

Hhip siRNA (sc-40164)	Sequence (5′→3′)
Set A	Sense: CCAUCGGCUCUUCAUUCUAtt
	Antisense: UAGAAUGAAGAGCCGAUGGtt
Set B	Sense: CGAACAAGUGCCUCUGUAAtt
	Antisense: UUACAGAGGCACUUGUUCGtt
Set C	Sense: CCAAACUCGAGGUUGUAUAtt
	Antisense: UAUACAACCUCGAGUUUGGtt

Fig. S2. The *in vitro* studies. (a) qPCR (*Nox2* and *Nox4* mRNA expression) in cells treated by rHhip (0-5ng/ml). (b) NADPH activity in cells treated by rHhip (0-5ng/ml); (c-d) qPCR (c, *Nox 2 mRNA*; d, *Nox4 mRNA*) in cells treated by PA (0.3mM). (e) qPCR (Hhip-siRNA efficiency) and Hhip-siRNA sequences. Three to four separated experiments; Data shown as mean ± SEM; 1 way-ANOVA followed by Bonferroni's post hoc test; *$p \le 0.05$; ***$p \le 0.001$; NS, non-significant vs INS-1 832/13 cells cultured in medium with 0.5% BSA (100%).

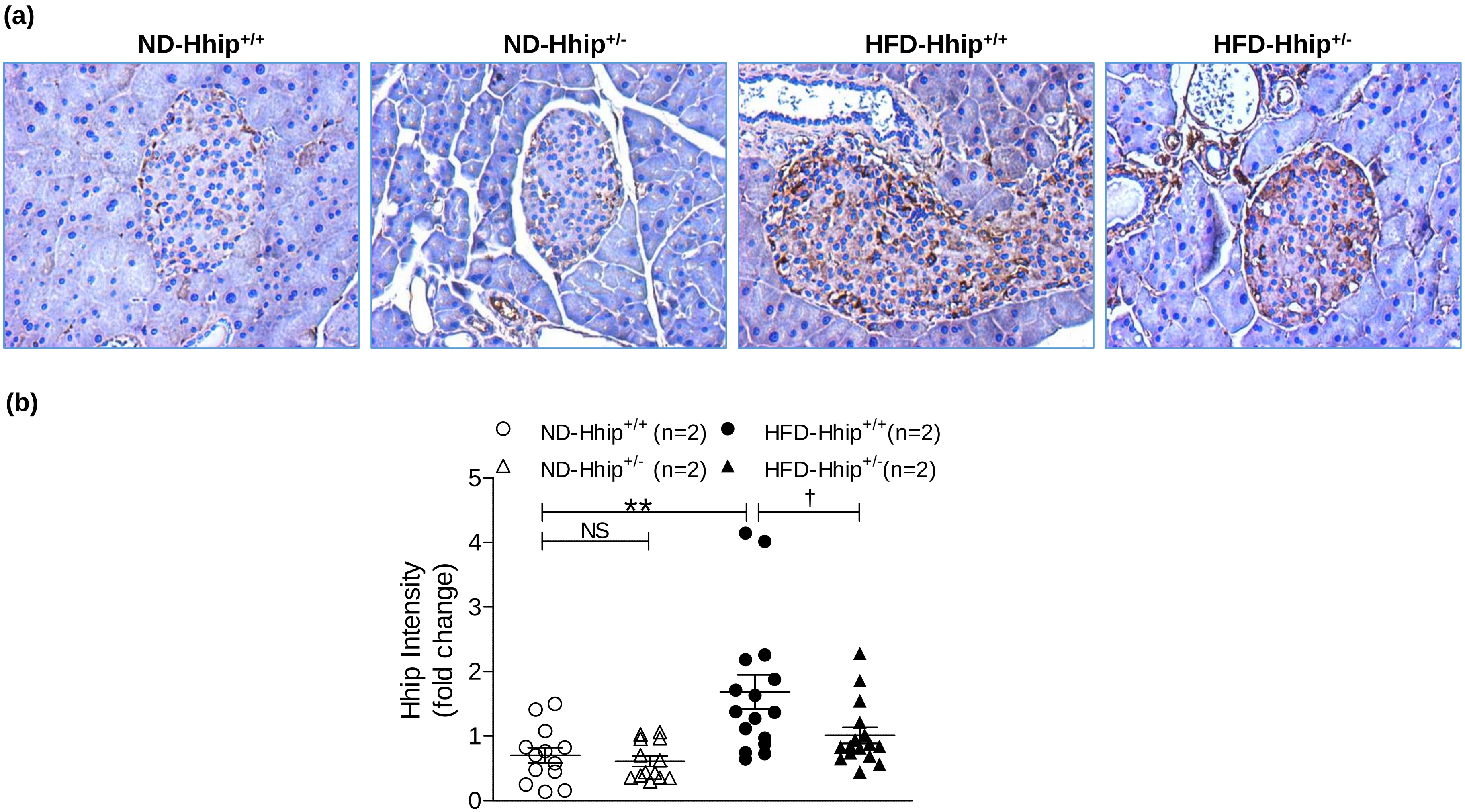

Fig. S3. Hhip–IHC in the islets among 4 subgroups of female mice (Hhip $^{+/+}$ vs Hhip $^{+/-}$; ND vs HFD; n=2/group) at 14 week-old. (b) Semi-quantification of staining; ND-Hhip $^{+/+}$ (n=2, 13 islets); ND-Hhip $^{+/-}$ (n=2, 13 islets); HFD-Hhip $^{+/+}$ (n=2, 16 islets); HFD-Hhip $^{+/-}$ (n=2, 16 islets); Data shown as mean ± SEM; 1 way-ANOVA followed by Bonferroni's post hoc test. *p ≤0.05; ** $p \le 0.01$ vs. ND-Hhip$^{+/+}$; $^{\dagger}p \le 0.05$ HFD-Hhip $^{+/+}$ vs. HFD-Hhip $^{+/-}$; NS, non-significant.
